# Educational technostress in Andean South America: regional evidence shaping digital wellbeing agenda for young adults

**DOI:** 10.3389/fpubh.2025.1724479

**Published:** 2026-01-12

**Authors:** Alejandro Vega-Muñoz, Joan Boada-Grau, Beatriz Sora, Natalia Salas-Guzmán, Nicolás Contreras-Barraza

**Affiliations:** 1 Facultad de Medicina y Ciencias de la Salud, Universidad Central de Chile, Santiago, Chile; 2 Facultad de Ciencias Empresariales, Universidad Arturo Prat, Santiago, Chile; 3 Faculty of Education Sciences and Psychology, University Rovira i Virgili, Tarragona, Spain; 4 Facultad de Educación y Ciencias Sociales, Universidad Finis Terrae, Santiago, Chile; 5 Facultad de Ciencias Económicas y Administrativas, Pontificia Universidad Católica de Valparaíso, Valparaíso, Chile

**Keywords:** Andean South America, digital wellbeing, educational policy educational technostress, psychological adjustment, young adults

## Abstract

Technostress affects the mental health of young adults in digitized educational environments, especially in contexts of high demands and low institutional support. Empirical evidence from Andean South America reveals digital overload, technological anxiety, and exhaustion among university students, teachers, and administrators. Lack of regulation and adaptive training exacerbates technostress, affecting psychological adjustment and quality of life. This policy brief proposes a regional roadmap to mitigate technostress through organizational redesign, psychometric assessment, and digital wellbeing policies. Concrete recommendations are presented for governments, educational communities, and employers.

## 1 Introduction

Technostress has become a silent epidemic in Andean higher education, undermining learning and wellbeing among students and faculty. Digital hyperconnectivity has expanded access, collaboration, and flexibility, reshaping teaching and institutional practices (1), yet intensive and unregulated use generates anxiety, overload, frustration, and exhaustion, harming emotional health and performance (2, 3). Conceptualized as a structural negative interaction normalized by constant availability (4, 5), it is classically defined as an adverse psychological response affecting motivation, health, and productivity (6). Covid-19 digital surge intensified these impacts, with work-from-home, monitoring, privacy risks, and socioeconomic inequalities amplifying technostress (7).

This policy brief draws on empirical evidence from Andean South America identified through a systematic search in Scopus, chosen for its broad regional coverage and parallel indexing in Web of Science Core Collection (WoSCC). The query {TITLE-ABS-KEY(technostress)} was restricted to 2000–2025. We reviewed documents referencing Andean countries (Argentina, Bolivia, Chile, Colombia, Ecuador, Peru) in educational contexts. Phase one identified 17 articles; phase two, through citation analysis, expanded the corpus to 23; and phase three, citation tracking, yielded a final set of 27 studies forming the empirical foundation of this brief (details in Supplementary material).

Technostress in Andean region is extensively documented through validated tools and empirical studies. Chilean teachers reported fatigue, anxiety, or both, with telework intensifying stress (8, 9). Peruvian students showed high and very high levels (10), while Ecuadorian teachers revealed four RED-TIC dimensions (11). Monitoring capacity is strengthened by RED-TIC, TS4US, and Tele-Cov-19 (12, 13). Key drivers include communication overload (14), ICT demands (15), poverty (16), and telework reducing quality of life (QoL) (17).

Technostress in Andean region manifests as anxiety, fatigue, depression, inefficacy, and physical strain. Chilean teachers show gender differences, with men more affected by anxiety and women by fatigue (8, 9), while Peruvian women scored higher across dimensions (18). Colombian professors reported anxiety and depression (19), STEM Ecuadorian teachers experienced distress (20), and 98% of Chilean teachers suffered musculoskeletal disorders (21). Protective factors include leadership (22), digital competencies (23, 24), resilience (25), and ChatGPT workflows (26). Overall, technostress is multidimensional, shaped by overload, inequality, telework, and skill gaps, requiring systemic interventions to address poverty and institutional practices (15, 16).

## 2 Policy options and implications

Regional evidence identifies technostress in young adults as multidimensional and rooted in educational systems. Healthier digital environments demand policies addressing structural malaise, individual capacities, and organizational conditions. Peer-reviewed local studies (2020–2026) provide a contextualized roadmap for mitigating impacts within Andean region itself.

### 2.1 Option 1: incorporate digital wellbeing into education and labor policies

Technostress is an emerging psychosocial risk that undermines sustainable education. Evidence from Chile, Colombia, Peru, and Ecuador shows that excessive digital demands, poor work-family balance, and limited institutional support generate anxiety, fatigue, musculoskeletal disorders, reduced academic performance, and diminished QoL among teachers, administrators, and students (9, 12, 17, 19, 21, 27, 28). Studies highlight inequalities and protective factors such as resilience, digital competencies, and leadership, while recent evidence suggests AI can reduce fatigue (14, 22, 23, 25, 26). Coordinated policies are urgently needed (3).

### 2.2 Option 2: adaptive training in digital skills with a focus on mental health

Digital training must extend beyond technical literacy to include emotional, cognitive, and organizational management of digital workloads. Evidence from Chile, Colombia, Ecuador and Peru shows that technostress reduces wellbeing, academic performance, and QoL, while resilience, digital competencies, and leadership act as protective factors (9, 15, 17, 25). Programs should foster ethical inquiry, student agency, and collaboration (29, 30), ensuring equitable access regardless of resources (31, 32). Validated scales such as TS4US, RED-TIC, and Peruvian instruments enable contextual monitoring (10–12, 33, 34).

### 2.3 Option 3: organizational redesign to mitigate techno-creators and empower techno-inhibitors

Effective technological integration requires cultural change within institutions (35). Design of digital environments directly shapes emotional experiences, making it essential to reduce techno-creators and strengthen techno-inhibitors (36). Evidence from Colombia, Chile, Peru, and Ecuador highlights the need for policies on digital disconnection, ergonomics, and emotional support, given impacts such as anxiety, fatigue, musculoskeletal disorders, and poor work–family balance (15, 17, 19, 21, 37). Spontaneous coping strategies developed by teachers can be systematized as institutional good practices for digital wellbeing (16, 25).

**Figure 1:**
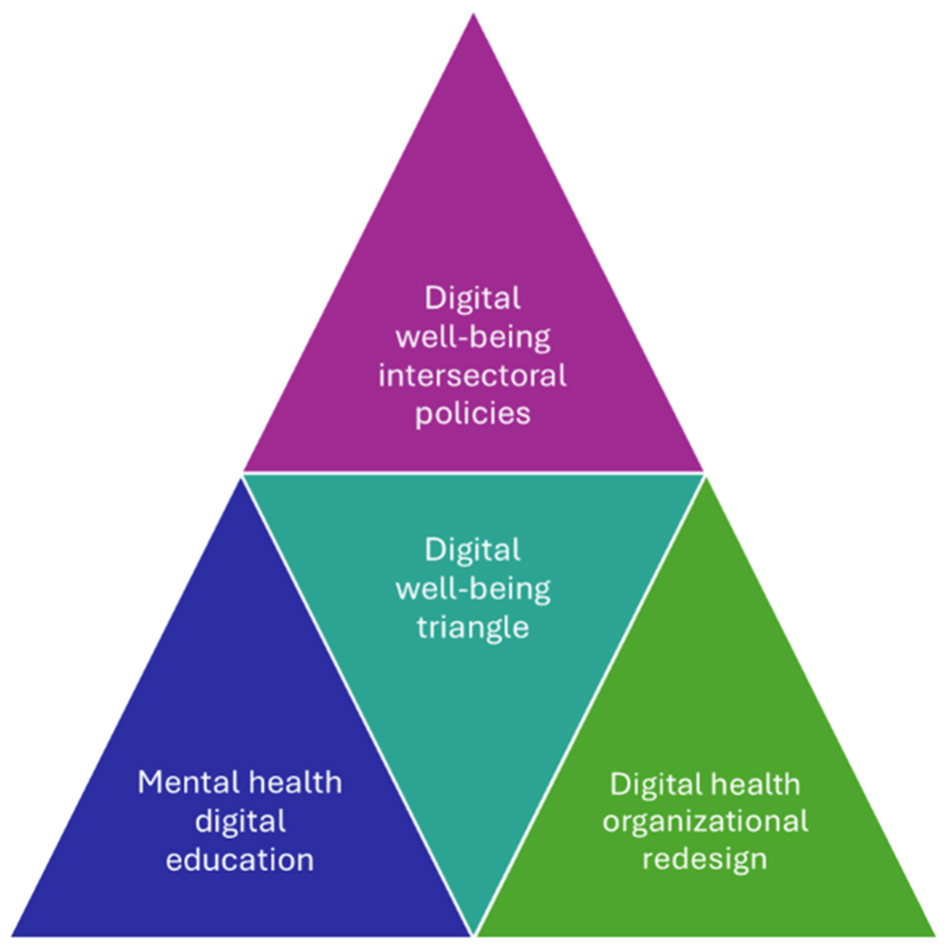
Digital wellbeing triangle.

Figure 1 presents the digital wellbeing triangle as a strategic framework integrating intersectoral policies, mental health education, and organizational redesign of digital environments. These three options align with SDGs 3 (health), 4 (education), and 8 (decent work), and must be conceived as interdependent components of a comprehensive strategy (38). Recognizing technostress as a psychosocial risk, promoting adaptive training with a mental health focus, and redesigning institutional digital spaces are complementary actions. Their implementation can enhance psychological adjustment among young adults, strengthen institutional sustainability, and improve educational quality in digital society.

## 3 Actionable recommendations

Policy brief transforms technostress diagnosis into actionable measures for education and labor systems, recommending institutional, educational, and organizational interventions to strengthen young adults' digital wellbeing, grounded in Andean evidence and adapted to shared structural challenges (see Table 1).

**Table 1:** Political recommendations.

**Actor**	**Recommendation**
Ministries of education and labor	Recognizing technostress as an emerging psychosocial risk is essential to safeguard healthier education and work environments. Ministries should integrate it into national mental health assessments, occupational health protocols, and regulatory frameworks, updating regulations and generating digital well-being indicators. Establish a National Digital Wellbeing Observatory to monitor technostress, guide policy updates, and coordinate intersectoral actions across education, health, and labor.
Educational institutions	Recognizing technostress as a psychosocial risk is key to improving mental health and academic performance. Institutions should integrate it into diagnoses, improvement plans, and well-being protocols, using validated scales such as TS4US, RED-TIC, and Andean instruments to assess digital well-being. A practical step is to create systematic monitoring units that apply these tools regularly, enabling preventive and adaptive interventions grounded in local evidence.
Schools and universities	Designing digital training programs with a psychosocial focus enables students and educators to manage emotional demands and digital overload. Institutions should reduce techno-creators and strengthen techno-inhibitors such as autonomy and emotional support. Implementation requires institutionalize digital well-being programs that integrate coping strategies, resilience-building, and ergonomic policies to foster healthier learning environments.
Employers	Implementing digital disconnection policies, ergonomic practices, and emotional support helps remote workers manage technostress and sustain mental well-being. Reducing techno-creators like constant availability and overload, while strengthening techno-inhibitors such as autonomy and recognition, fosters healthier workplaces. An effective mechanism would be institutional well-being programs that combine ergonomic training, recognition systems, and clear disconnection protocols to prevent burnout and promote sustainable digital cultures in hybrid or remote organizations.

These recommendations outline pathways to create humane, sustainable, and emotionally safe digital environments. Their effectiveness relies on political will, institutional leadership, and cross-sectoral collaboration. Properly implemented, they strengthen mental health, resilience, and educational quality, affirming digital wellbeing as essential for learning and decent work.

## 4 Conclusions

Technostress is a structural weakness in digital systems, undermining mental health in education and obstructing adjustment, motivation, and learning (3, 6, 20). Evidence from Andean Region highlights hyperconnectivity, limited support, and socioeconomic inequalities, urging policies to redesign environments, regulate telework, and implement psychosocial training (34, 39). Psychometric studies in Peru confirm failed adaptation to hyperconnectivity, offering monitoring tools (10, 12). Future research should examine how regulation and ethics mitigate generative AI threats (40).

## Supplementary material

Scientific evidence on educational technostress in Andean South America is presented in Supplementary Table 1 and dataset: PB1137.xlsx.
