# Supplementary material for: Educational technostress in Andean South America: regional evidence shaping digital wellbeing agenda for young adults
Source: Front Public Health. 2026 Jan 12;13:1724479. doi: 10.3389/fpubh.2025.1724479 (PMC12832322; doi:10.3389/fpubh.2025.1724479)
Supplement: Supplementary file 2 [file Table_1.docx]

Supplementary Material

# Supplementary Table 1. Scientific Evidence on Educational technostress in Andean South America.

This table compiles 27 studies on educational technology stress from Colombia (4), Chile (5), Ecuador (5), and Peru (13), providing supplementary evidence that underpins the recommendations presented in this policy brief.

| Count | Phase | ID | Authors | Year | Source title | Cited by | DOI* | EID** | ADD |
| --- | --- | --- | --- | --- | --- | --- | --- | --- | --- |
| 1 | 1 | 1 | Estrada-Muñoz, C.; Castillo, D.; Vega-Muñoz, A.; Boada I Grau, J. | 2020 | International Journal of Environmental Research and Public Health | 46 | 10.3390/ijerph17155280 | 2-s2.0-85088285865 | ADD01 |
| 2 | 1 | 2 | Álvarez-Risco, A.; Del-Aguila-Arcentales, S.; Yáñez, J.A.; Rosen, M.A.; Mejia, C.R. | 2021 | Sustainability (Switzerland) | 65 | 10.3390/su13168949 | 2-s2.0-85112420199 | ADD02 |
| 3 | 1 | 3 | Estrada-Muñoz, C.; Vega-Muñoz, A.; Castillo, D.; Muller-Pérez, S.; Boada I Grau, J. | 2021 | International Journal of Environmental Research and Public Health | 55 | 10.3390/ijerph18105458 | 2-s2.0-85106144957 | ADD03 |
| 4 | 1 | 4 | Urbano, O.F.A.; Gabriel Elías Chanchí, G.G.E.; Campo, M.W.Y. | 2021 | TEM Journal | 1 | 10.18421/TEM104-22 | 2-s2.0-85121844047 | ADD04 |
| 5 | 1 | 5 | Reyes, R.; Libaque-Sáenz, C.F. | 2023 | Issues in Information Systems | 2 | 10.48009/4_iis_2023_114 | 2-s2.0-85174247256 | ADD05 |
| 6 | 1 | 6 | Espíritu-Romero, Y.; Iraola-Real, I. | 2023 | RISTI - Revista Iberica de Sistemas e Tecnologias de Informacao | 1 |  | 2-s2.0-85162918477 | ADD06 |
| 7 | 1 | 7 | Andrade, J.M.; Ramírez Plazas, E.R.; Ramírez, J.C.; Castro, D.B. | 2023 | Problems and Perspectives in Management | 1 | 10.21511/ppm.21(4).2023.36 | 2-s2.0-85179834054 | ADD07 |
| 8 | 1 | 8 | Ortega-Jiménez, D.; Angelucci Bastidas, L.T.; López-Guerra, V.M.; Ramírez, M.R. | 2023 | Psychology Research and Behavior Management | 4 | 10.2147/PRBM.S436047 | 2-s2.0-85179354139 | ADD08 |
| 9 | 1 | 9 | Vásquez-Pajuelo, L.; Rodriguez-Barboza, J.R.; Bartra-Rivero, K.R.; Andrade-Díaz, E.M.; Tuesta-Vila, J.A.; Obando-Peralta, E.C.; Alarcón-Villalobos, Y.J. | 2024 | Journal of Ecohumanism | 2 | 10.62754/joe.v3i4.3339 | 2-s2.0-85202553852 | ADD09 |
| 10 | 1 | 10 | Bartra-Rivero, K.R.; Vásquez-Pajuelo, L.; Avila-Sánchez, G.A.; Andrade-Díaz, E.M.; Mendez-Ilizarbe, G.S.; Rodriguez-Barboza, J.R.; Alarcón-Villalobos, Y.J. | 2024 | Data and Metadata | 6 | 10.56294/dm2024303 | 2-s2.0-85193239714 | ADD10 |
| 11 | 1 | 11 | Herrera-Sánchez, M.J.; Casanova-Villalba, C.I.; Moreno-Novillo, Á.C.; Mina-Bone, S.G. | 2024 | Revista Venezolana de Gerencia | 0 | 10.52080/rvgluz.29.e11.36 | 2-s2.0-85198850345 | Non data |
| 12 | 1 | 12 | Cajas-Bravo, V.T.; Huanca-Rojas, L.M.; Arias Lizares, A.; Ramírez Cajamarca, J.C.; Vasquez Perdomo, F.; de la Cruz Cruz, M.A.; Romero Giron, H.; Guerrero Millones, A.M.; Dávila-Morán, R.C. | 2025 | Education Sciences | 0 | 10.3390/educsci15091223 | 2-s2.0-105017810834 | Non data |
| 13 | 1 | 13 | Araya-Ugarte, G.; Armesto-Céspedes, M.; Contreras-Barraza, N.; Vega-Muñoz, A.; Salazar-Sepúlveda, G.; Nelson David, N. | 2025 | Sustainability (Switzerland) | 0 | 10.3390/su17156974 | 2-s2.0-105013348598 | Non data |
| 14 | 1 | 14 | Estrada-Araoz, E.G.; Cruz-Laricano, E.O.; Gallegos-Ramos, N.A.; Manrique-Jaramillo, Y.V.; Yabar-Miranda, P.S.; Achata-Cortez, C.A. | 2025 | Gaceta Medica de Caracas | 1 | 10.47307/GMC.2025.133.1.15 | 2-s2.0-105000548665 | ADD14 |
| 15 | 1 | 15 | Orosco-Fabian, J.R.; Pomasunco-Huaytalla, R.; Gómez-Galindo, W.; Torres-Cortez, E.E. | 2025 | Revista Colombiana de Educacion | 0 | 10.17227/rce.num94-18243 | 2-s2.0-85214382869 | Non data |
| 16 | 1 | 16 | Verde-Avalos, E.; Turpo-Chaparro, J.E.; Palomino-Ccasa, J.; Requena-Cabral, G.; Malca-Peralta, S.S. | 2025 | Frontiers in Psychology | 1 | 10.3389/fpsyg.2025.1503442 | 2-s2.0-105001481136 | ADD16 |
| 17 | 1 | 17 | Cataldo, A.; Bravo-Adasme, N.; Riquelme, J.; Vásquez, A.; Rojas, S.; Arias-Oliva, M. | 2025 | International Journal of Environmental Research and Public Health | 1 | 10.3390/ijerph22070986 | 2-s2.0-105011619792 | ADD17 |
| 18 | **2** | ADD03.1 | Choconta, J.; Pineda-Báez, C.; J. | 2026 | International Journal of Educational Research Open | 0 | 10.1016/j.ijedro.2025.100566 | 2-s2.0-105022893372 | Non data |
| 19 | 2 | ADD03.2 | Navarro-Espinosa, J.A.; Vaquero Abellán, M.; Perea-Moreno, A.-J.; Pedrós-Pérez, G.; Aparicio-Martínez, P.; Martínez-Jiménez, M.P. | 2021 | International Journal of Environmental Research and Public Health | 25 | 10.3390/ijerph18189605 | 2-s2.0-85114609445 | ADD18 |
| 20 | 2 | ADD03.3 | Vega-Fernandez, G.; Gonzalez-Torres, C.; Solís-Soto, M.; Lizana, P.A. | 2024 | Frontiers in Public Health | 2 | 10.3389/fpubh.2024.1277578 | 2-s2.0-85193483679 | ADD19 |
| 21 | 2 | ADD03.4 | Lizana, P.A.; Vega-Fernadez, G. | 2021 | International Journal of Environmental Research and Public Health | 79 | 10.3390/ijerph18147566 | 2-s2.0-85110511103 | ADD20 |
| 22 | 2 | ADD06.1 | Herrera-Sánchez, M.J.; Casanova-Villalba, C.I.; Moreno-Novillo, Á.C.; Mina-Bone, S.G. | 2024 | Revista Venezolana de Gerencia | 0 | 10.52080/rvgluz.29.e11.36 | 2-s2.0-85198850345 | Non data |
| 23 | 2 | ADD07.1 | Mallma-Canchaya, Á.W. | 2025 | Educacion y Humanismo | 0 | 10.17081/eduhum.27.49.7514 | 2-s2.0-105014112419 | Non data |
| 24 | 3 | ADD18.1 | Hermosa-Bosano, C.; Paz, C.; Hidalgo-Andrade, P. | 2024 | Trends in Psychology | 1 | 10.1007/s43076-022-00228-w | 2-s2.0-85137218194 | ADD21 |
| 25 | 3 | ADD20.1 | Calvo-Paz, M.; Guevara-Ramírez, J.; Zapata-López, J.S.; Realpe-Martínez, D.L. | 2022 | Brazilian Journal of Occupational Therapy | 0 | 10.1590/2526-8910.CTOAO247832423 | 2-s2.0-85143203758 | Non data |
| 26 | 3 | ADD20.2 | Jurado-Enríquez, E.; Vargas-Prado, K.; Jurado-Retamoso, P. | 2023 | Human Review. International Humanities Review / Revista Internacional de Humanidades | 0 | 10.37467/revhuman.v18.4855 | 2-s2.0-85149801340 | Non data |
| 27 | 3 | ADD20.3 | Mamani-Benito, O.; Quispe, E.J.S.; Jallo, L.Y.; Carranza Esteban, R.F.C.; Turpo-Chaparro, J.E.T.; Lingán-Huamán, S.K. | 2022 | Archivos de Prevencion Riesgos Laborales | 3 | 10.12961/aprl.2022.25.03.03 | 2-s2.0-85140284263 | ADD22 |

*DOI: Digital Object Identifier, **EID: Electronic Identifier (Scopus).
